# Sphericity of lymph nodes using 3D-CT predicts metastasis in lung cancer patients

**DOI:** 10.1186/s40644-023-00635-x

**Published:** 2023-12-17

**Authors:** Kazuto Sugai, Yasuharu Sekine, Tomoyuki Kawamura, Takahiro Yanagihara, Yusuke Saeki, Shinsuke Kitazawa, Naohiro Kobayashi, Shinji Kikuchi, Yukinobu Goto, Hideo Ichimura, Yukio Sato

**Affiliations:** 1 Department of Thoracic Surgery, University of Tsukuba, 1-1-1 Tennodai, Tsukuba, 305-8575 Ibaraki Japan; 2 Ibaraki Prefectural Hospital, 6528, Koibuchi, Kasama, 309-1793 Ibaraki Japan

**Keywords:** Lymph node Metastasis, Lung cancer, Computer tomography, Three-dimensional computer tomography, Diagnosis, Sphericity

## Abstract

**Background:**

The presence of mediastinal lymph node metastasis is important because it is related to the treatment and prognosis of lung cancer. Although prevalently used, evaluation of lymph nodes is not always reliable. We introduced sphericity as a criterion for evaluating morphologic differences between metastatic and nonmetastatic nodes.

**Methods:**

We reviewed the cases of 66 patients with N2 disease and of 68 patients with N0-1 disease who underwent lobectomy with mediastinal dissection between January 2012 and December 2021. The sphericity of the dissected station lymph nodes, which represents how close the node is to being a true sphere, was evaluated along with the diameter and volume. Each parameter was obtained and evaluated for ability to predict metastasis.

**Results:**

Metastatic lymph nodes had a larger short-axis diameter (average: 8.2 mm vs. 5.4 mm, p < 0.001) and sphericity (average: 0.72 vs. 0.60, p < 0.001) than those of nonmetastatic lymph nodes. Short-axis diameter ≥ 6 mm and sphericity ≥ 0.60 identified metastasis with 76.2% sensitivity and 70.2% specificity (AUC = 0.78, p < 0.001) and 92.1% sensitivity and 53.9% specificity (AUC = 0.78, p < 0.001), respectively. For lymph nodes with a short-axis diameter ≥ 5 mm, sphericity ≥ 0.60 identified metastasis with 84.1% sensitivity and 89.3% specificity.

**Conclusion:**

By using 3D-CT analysis to examine sphericity, we showed that metastatic lymph nodes became spherical. Our method for predicting lymph node metastasis based on sphericity of lymph nodes with a short-axis diameter ≥ 5 mm could do so with higher sensitivity than the conventional method, and with acceptable specificity.

## Background

Lung cancer is the leading cause of cancer death despite advances in treatment, and its prognosis remains insufficient. Nodal staging is an important factor in predicting prognosis and deciding appropriate treatments. Computed tomography (CT) has been used as the initial imaging modality for diagnosis and for staging of lymph node metastasis. The size criterion using CT—ie, a lymph node with a short-axis diameter greater than or equal to 1 cm is regarded as metastasis [1]—has been mostly used since the 1980s for predicting mediastinal lymph node metastasis. The current guidelines (American Thoracic Society, American College of Clinical Pharmacy, and European Society of Thoracic Surgery) recommend CT for preoperative nodal evaluation. However, these guidelines describe the size criterion only in terms of the short-axis diameter of lymph nodes shown on CT and, therefore, did not provide the required accuracy [2–4].

The short-axis diameter of relatively small metastatic lymph nodes could be smaller than 1 cm, which explains the limited sensitivity of the size criteria. On the other hand, it was reported that metastatic lymph nodes tended to be more spherical than nonmetastatic ones [5], indicating that in addition to the size, the shape of lymph nodes is important in predicting metastasis. Regarding size, in addition to the prevalently used short-axis diameter, volume was reported as a useful predictor of metastasis [6]. Regarding shape, the short-axis/long-axis ratio established for superficial lymph nodes using ultrasound sonography was reported in a study of CT of lung cancer patients [7–9]. However, the reported short-axis/long-axis ratio obtained on CT was analyzed using only axial sections. Because lymph nodes are three-dimensional (3D) objects and could be irregularly shaped in any direction, analysis using only axial sections could miss the features of a lymph node. Therefore, 3D-CT analysis could evaluate lymph node morphology more precisely. We evaluated lymph node morphology using 3D-CT and introduced a novel index, sphericity, to evaluate the shape of lymph nodes sterically.

The aim of the present study was to evaluate the efficacy of 3D morphologic analysis to predict lymph node metastasis. The sphericity and volume of lymph nodes were evaluated using 3D-CT analysis. Their efficacies were compared with those evaluated using 2D-CT analysis, short-axis diameter, and the short-axis/long-axis ratio.

## Methods

### Study population

**Fig. 1:**
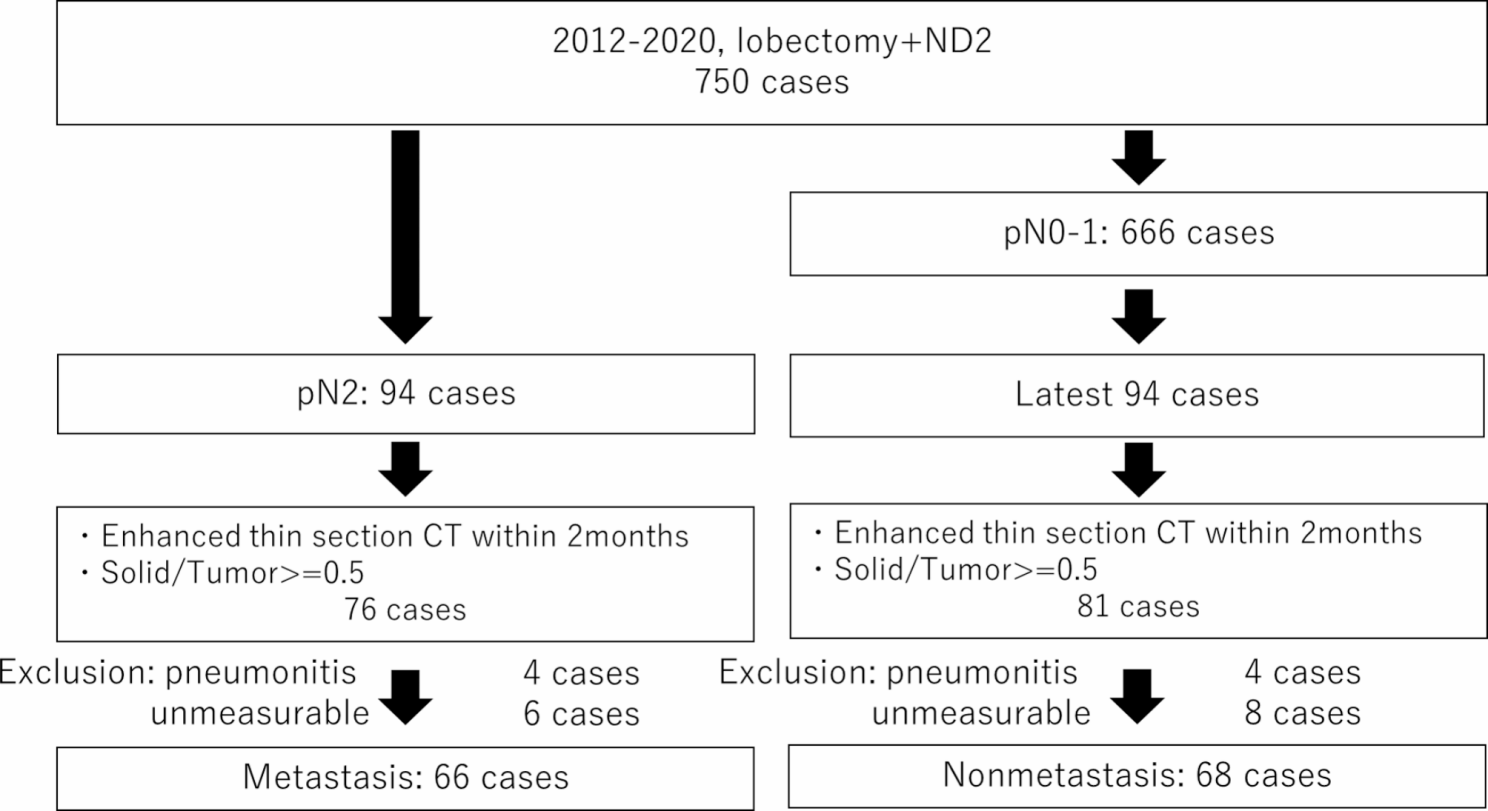
Procedure for extraction of a target lymph node. Figure **A:** representative CT image from the metastatic group with peritracheal nodal metastasis. Figure **B:** the target lymph node was automatically separated by manual dragging of the long-axis diameter. Figure **C** and **D:** extracted target lymph node seen from the axial and coronal CT planes

Between January 2012 and December 2021, 750 patients underwent pulmonary lobectomy or pneumonectomy with mediastinal lymph node dissection at the University of Tsukuba Hospital. Among them, 94 patients had pathologically proven N2 disease, and 666 patients had pathologically proven N0 or N1 disease (mediastinal node negative). To match the number of the mediastinal node-negative group with that of the node-positive group, the latest 94 N0-1 patients were included. The patients with thin-section contrast-enhanced CT scans within 2 months of the operation and with tumors with a consolidation/tumor ratio of ≥ 0.5 were evaluated. The consolidation/tumor ratio was defined as the ratio of the diameter of consolidation to the total diameter of the tumor in the lung field. The consolidation area was defined as an area of increased opacification that completely obscured the underlying vascular signs. We analyzed the tumors with a consolidation/tumor ratio ≥ 0.5 because tumors with a consolidation/tumor ratio < 0.5 are generally indolent, noninvasive, and less likely to have lymph node metastasis [10]. Patients with obstructive pneumonia (4 cases in both groups) were excluded. Patients whose lymph nodes were too small (almost less than 3 mm) to be recognized with 3D-CT software (6 patients in the metastatic group and 8 patients in the nonmetastatic group) were also excluded. As a result, the remaining 66 patients of the metastatic group and 68 patients of the nonmetastatic group were analyzed (Fig. 1).

The medical records of all the patients were reviewed for age, sex, past medical history, and smoking index. The study was conducted according to the principles of the Declaration of Helsinki. Ethical approval for the study was obtained from the institutional review board of the University of Tsukuba Hospital (approval number R01-287). All the data in this retrospective study were anonymized, and informed consent from the patients was obtained using the opt-out method with a disclosure document.

### CT examination and lymph node measurement

High resolution CT images were obtained with multidetector-row CT scanners (Brilliance 64 and Brilliance iCT 256; Philips Electronics, Eindhoven, the Netherlands). A standard contrast-enhanced scanning protocol was performed to evaluate from the lung apex to the diaphragm using the following parameters: 120 kilovoltage peak, 180–280 mAs; resolution, 512 × 512 pixels; and scanning duration, 0.5 s. Axial images were reconstructed with a section thickness of 1 mm. Images were photographed using a window level of 30–60 HU with a window width of 350–600 HU (mediastinal window setting). All preoperative CT data were transferred to a computer workstation with 3D-CT software (SYNAPSE VINCENT version 3.0; FUJIFILM, Tokyo, Japan).

**Fig. 2:**
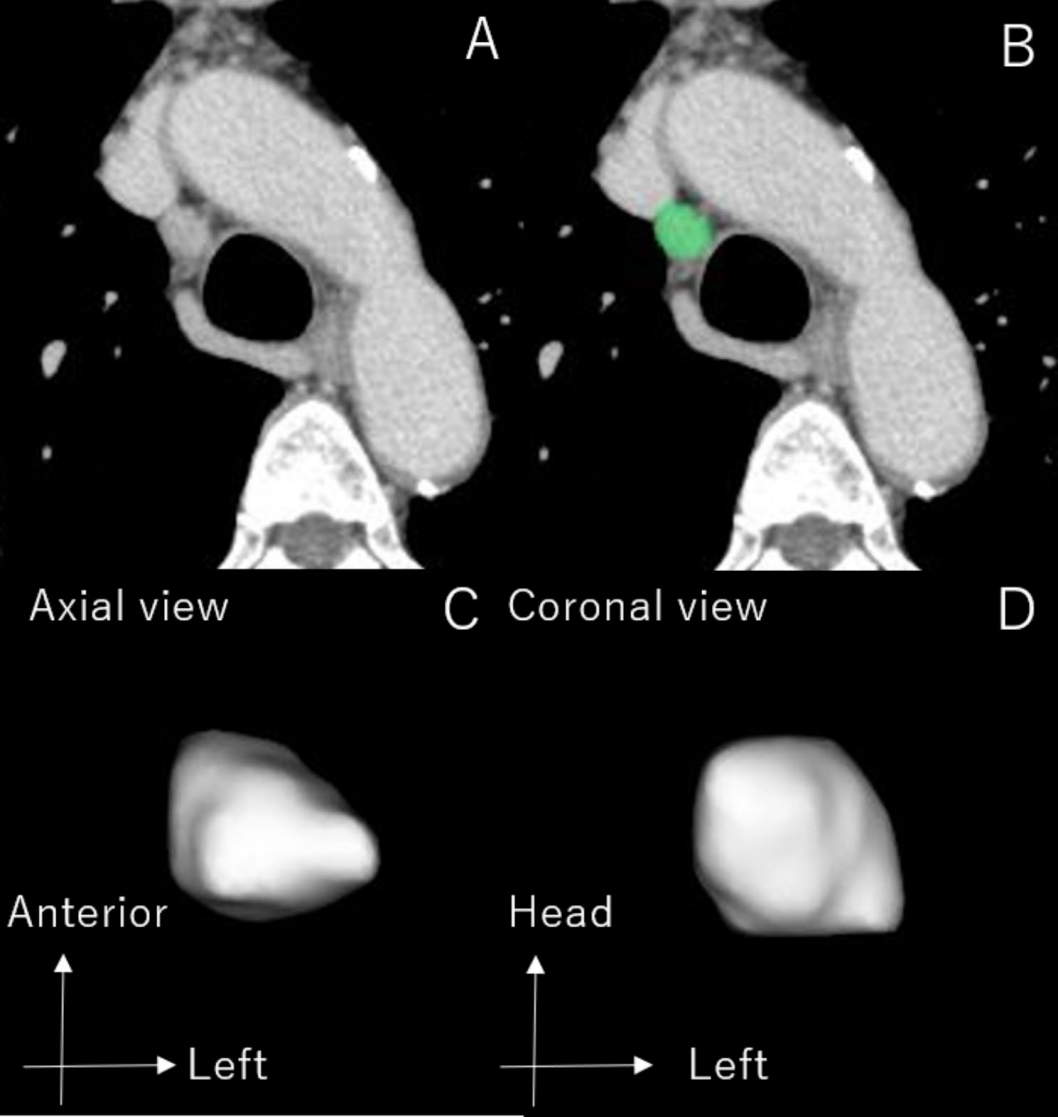
Schema of the study population. ND, nodal dissection

In the pathologically proven N2 group, lymph nodes of the pathologically positive stations were evaluated using CT. In the pathologically proven N0-1 group, lymph nodes of the pathologically negative mediastinal lymph nodes were evaluated. Each lymph node was evaluated for the short-axis and long-axis diameters and for the short-axis/long-axis ratio in the axial section as 2D analysis. Next, lymph nodes were extracted as 3D data by use of SYNAPSE VINCENT. For the extraction of 3D data, the observer tracked the longest diameters of the lymph nodes in the axial sections, and the imaging software semiautomatically detected the boundaries of whole lymph nodes. The extracted 3D data were used to measure the volume and the maximum diameter sterically and the surface area of the lymph nodes by use of the image analyzing software Fusion 360 with Netfabb® (Autodesk, San Francisco, CA, USA) (Fig. 2).

To evaluate how close the lymph node was to being a true sphere, we applied a sphericity index defined as [practical surface area] / [estimated surface area as a true sphere with the longest diameter] [11], in which a true sphere is indicated as 1. The estimated surface area was calculated as the surface area of the virtual true sphere with the longest diameter of the lymph node measured using 3D-CT. Therefore, lymph node sphericity was obtained with the following formula: Sphericity = [practical surface area] / {4π × [3d longest diameter / 2]^2^}.

### Statistical analysis

The groups were evaluated and compared for patient characteristics, short-axis diameter, volume, short-axis/long-axis ratio, and lymph node sphericity. Data were expressed as means ± standard deviations (SDs), and values for categorical variables were given as percentages. The χ^2^ test was used to compare categorical variables, and the Mann-Whitney U test, for nonparametric data. Receiver operating characteristic (ROC) curves for each parameter were constructed, and the area under the curve (AUC) was calculated with the histopathologic diagnosis as the outcome. All statistical analysis were performed using SPSS version 18.0 (IBM Corporation, Armonk, NY, USA). Probability values less than 0.05 were considered significant.

## Results

Table 1 shows the patient and tumor characteristics of the metastatic and nonmetastatic groups. Sixty-six patients were included in the metastatic group, and 68 patients, in the nonmetastatic group. No significant differences were found between the 2 groups in terms of age, sex, smoking index, or tumor diameter and histology.

**Table 1:** Patient characteristics of the metastatic and nonmetastatic groups

	Metastasis (n = 66)	Nonmetastasis (n = 68)	p-value
Age	68.9 ± 10.2	69.2 ± 6.8	0.646
Male / Female	39 / 24	45 / 23	0.611
Brinkmann Index	567 ± 548	684 ± 804	0.337
Interstitial pneumonia	1 (1.5%)	1 (1.5%)	0.974
Tumor diameter (mm)	34.6 ± 17.9	33.0 ± 21.2	0.628
Consolidation / Tumor ratio	0.99	0.96	0.002
Tumor histology			0.050
Adenocarcinoma	51 (78.5%)	40 (58.8%)	
Squamous carcinoma	10 (15.3%)	21 (30.9%)	
Other	4 (6.2%)	7 (10.3%)	

Table 2 shows the morphologic findings for the metastatic and nonmetastatic lymph nodes. The metastatic and nonmetastatic groups contained 63 and 141 lymph nodes, respectively. The short-axis diameter of the metastatic lymph nodes, 8.2 ± 3.7 mm, was larger than that of the nonmetastatic lymph nodes, 5.4 ± 1.7 mm (p < 0.001). The number of lymph nodes with a short-axis diameter ≥ 1 cm of the metastatic group, 13 of 63 (19.0%), was higher than that of the nonmetastatic group, 4/141 (2.8%). The short-axis/long-axis ratio of the metastatic lymph nodes, 0.68 ± 0.17, was larger than that of the nonmetastatic lymph nodes, 0.59 ± 0.15 (p = 0.001). The volume of the metastatic lymph nodes, 1679 ± 4509 mm^3^, was larger than that of the nonmetastatic lymph nodes, 552 ± 497 mm^3^ (p = 0.052). The sphericity of the metastatic lymph nodes, 0.72 ± 0.10, was larger than that of the nonmetastatic lymph nodes, 0.60 ± 0.10 (p < 0.001).

**Table 2:** Morphologic comparison of the metastatic and nonmetastatic lymph nodes

	Metastatic LN (n = 63)	Nonmetastatic LN (n = 141)	p-value
Short diameter (mm)	8.2 ± 3.7	5.4 ± 1.7	< 0.001
Short-axis / long-axis diameter	0.68 ± 0.17	0.59 ± 0.15	0.001
Volume (mm^3^)	1679 ± 4509	552 ± 497	0.052
Sphericity	0.72 ± 0.10	0.60 ± 0.10	< 0.001

LN, lymph node

**Fig. 3:**
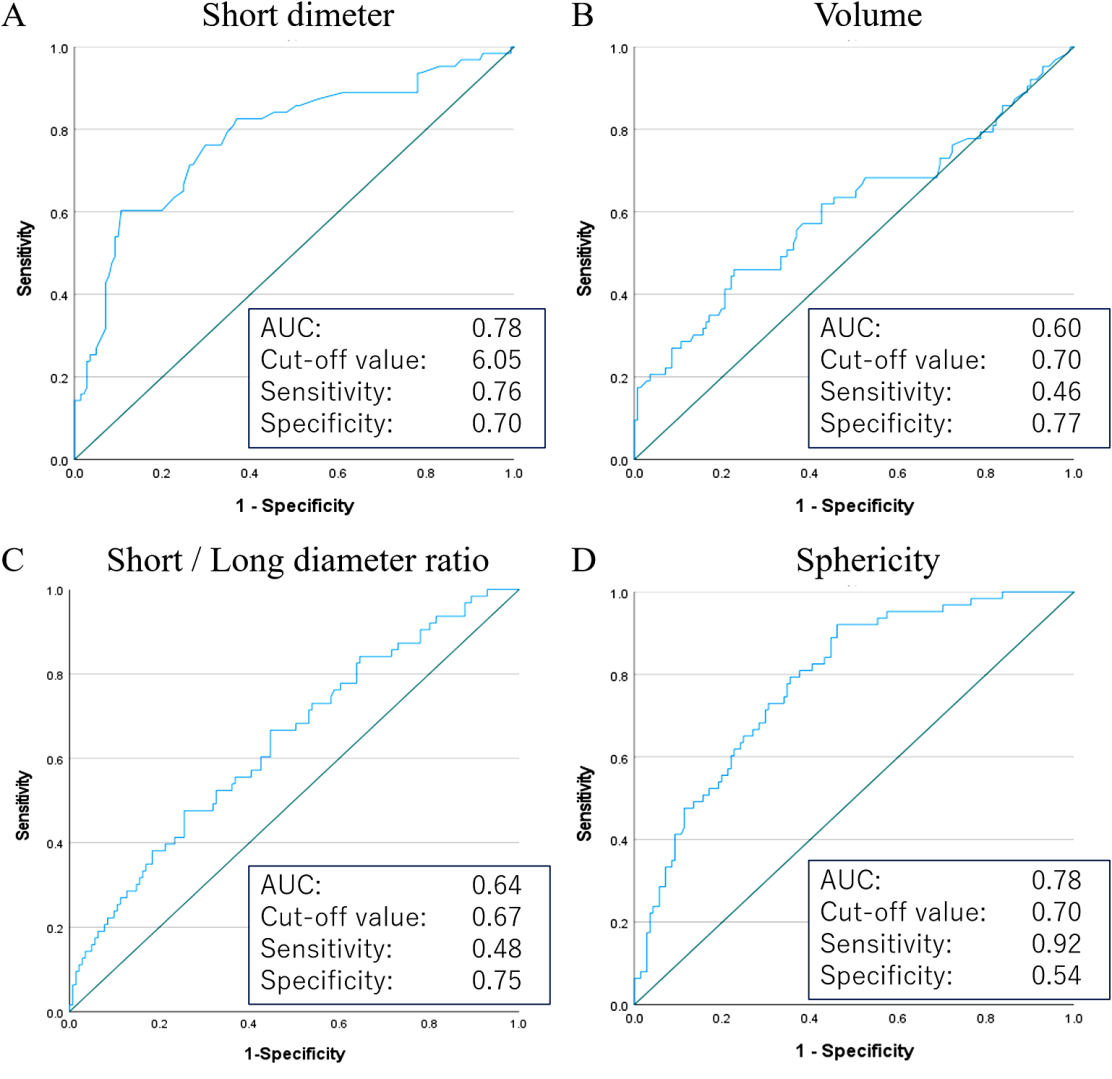
ROC curves for the prediction of malignancy using the (**A**) short-axis diameter, (**B**) volume, (**C**) short-axis/long-axis ratio, and (**D**) sphericity. The area under the ROC curve (AUC), optimal threshold, sensitivity, and specificity were provided

Figure 3 shows the ROC curve of the short-axis diameter (Fig. 3A), volume (Fig. 3B), short-axis/long-axis ratio (Fig. 3C), and sphericity (Fig. 3D) for sensitivity and specificity in predicting lymph node metastasis. ROC curve analysis of the short-axis diameter revealed that the optimal cutoff value for predicting metastatic lymph nodes was 6.0 mm and the sensitivity and specificity were 76.2% and 70.2%. The AUC of the short-axis diameter was 0.78. The ROC curve analysis of the volume revealed that the cutoff value was 0.70 and the sensitivity and specificity were 46.0% and 77.0%. The AUC of the volume was 0.60. The ROC curve analysis of the short-axis/long-axis ratio revealed that the cutoff value was 0.67 and the sensitivity and specificity were 48.0% and 75.0%. The AUC of the short-axis/long-axis ratio was 0.64. ROC curve analysis of the sphericity revealed that the cutoff value was 0.70 and the sensitivity and specificity were 92.1% and 53.9%. The AUC of the sphericity was 0.78.

**Fig. 4:**
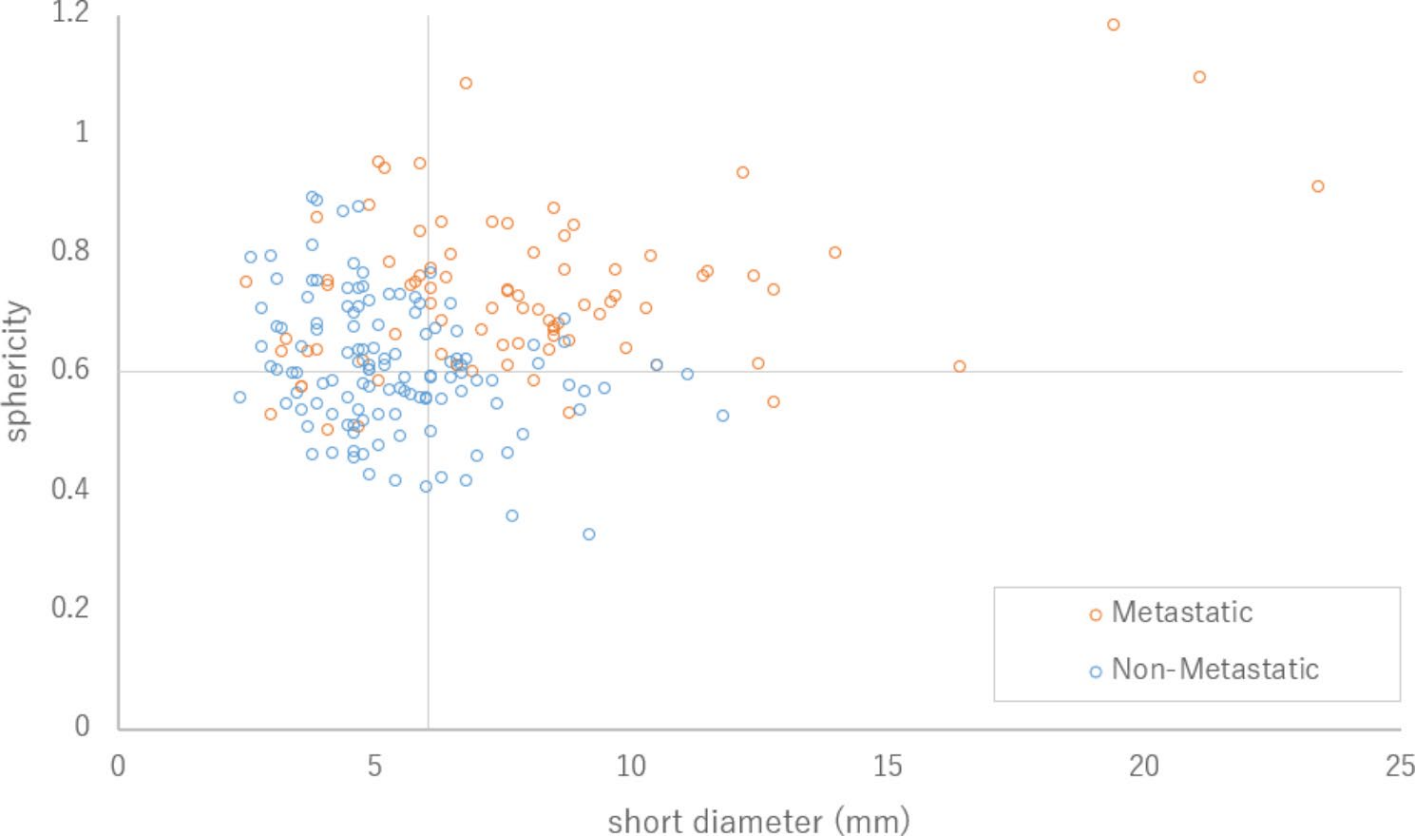
Scatterplot of the relationship between short-axis diameter and sphericity stratified by metastasis and nonmetastasis. The additional lines on the vertical and horizontal axes indicate the cutoff values for metastasis prediction obtained from the ROC curves

**Fig. 5:**
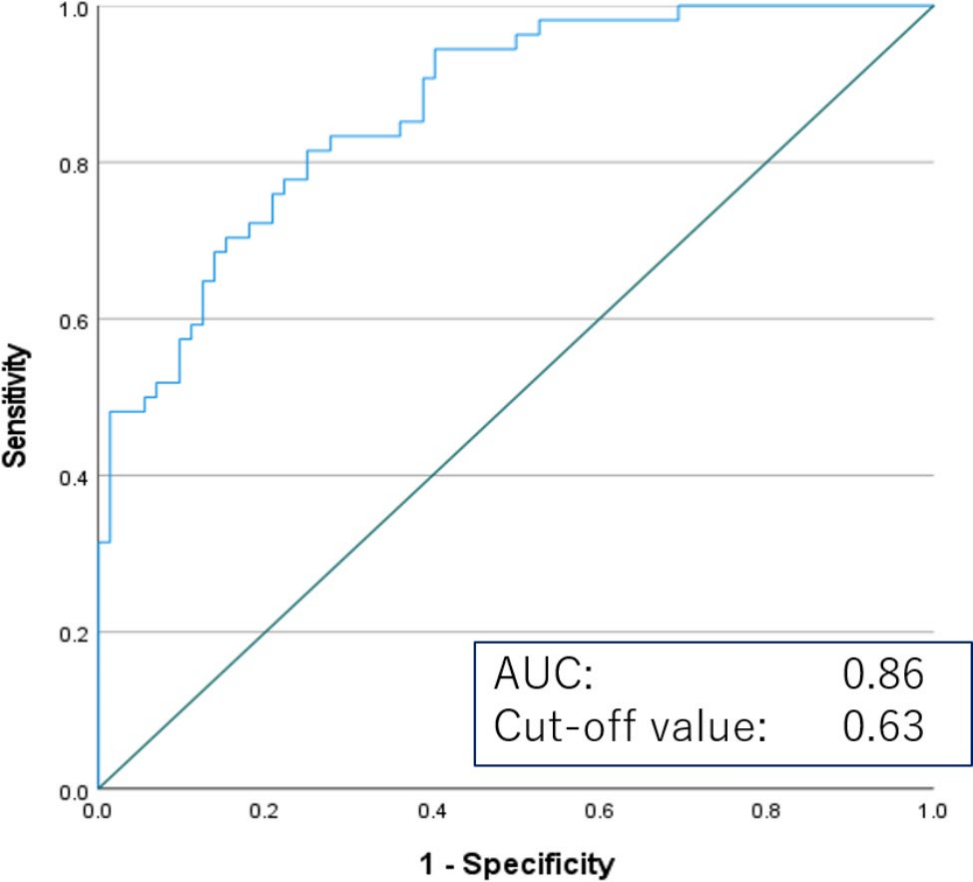
ROC curves for the prediction of malignancy using the sphericity among patients with lymph nodes with short-axis diameter ≥ 5 mm. The AUC and optimal threshold are provided

Figure 4 shows the scatterplot of the relationship of the short-axis diameter and sphericity with lymph node metastasis. Most of the relatively small lymph nodes, less than 5 mm in the short-axis diameter, were not metastatic regardless of the sphericity. Conversely, in lymph nodes with a short-axis diameter of 5 mm or greater, metastasis and nonmetastasis tended to be separated according to the sphericity. To avoid inaccuracy of the sphericity analysis in relatively small lymph nodes, ROC curve analysis was conducted for lymph nodes with short-axis diameters greater than or equal to 4 mm, 5 mm, or 6 mm (Table 3). When the lymph nodes with short-axis diameters ≥ 5 mm were analyzed, the sensitivity and specificity of sphericity were 84.1% and 89.3%, which were both considered to be satisfactory for clinical judgement. The AUC for sphericity of lymph nodes with a short axis diameter ≥ 5 mm was 0.86 (Fig. 5).

**Table 3:** Optimal sphericity cutoff values for each of the receiver operating characteristic (ROC) curves for lymph nodes with a short-axis diameter ≥ 4 mm, 5 mm, or 6 mm, as well as the sensitivities and specificities of those values

Short-axis diameter	Sphericity cut-off value	Sensitivity	Specificity
4 mm	0.63	76.1%	77.3%
5 mm	0.63	84.1%	89.3%
6 mm	0.67	53.9%	97.8%

## Discussion

This study investigated the morphologic differences of metastatic and nonmetastatic mediastinal lymph nodes by use of 2D- and 3D-CT analysis in patients with non-small cell lung cancer. The 3D-CT analysis of sphericity revealed that metastatic lymph nodes became closer to being spherical than did nonmetastatic lymph nodes. The ROC curves showed a higher AUC for the short-axis diameter and sphericity than for the short-axis/long-axis ratio and volume of lymph nodes. In addition, sphericity showed high sensitivity and specificity when applied to lymph nodes with a short-axis diameter of 5 mm or greater.

It was reported that most of the metastatic lymph nodes had a short-axis diameter greater than 1 cm [12], and this conventional size criterion has been widely applied for imaging evaluation of lymph node metastasis. Although measuring the short-axis diameters of lymph nodes is simple and practical, the accuracy of applying the short-axis diameter for the diagnosis of lymph node metastasis has decreased when compared with when it was first reported. A meta-analysis of studies from 2005 to 2015 reported that the sensitivity and specificity of the size criterion were 59% and 78%, respectively [1], which were both unsatisfactory. In our study, the sensitivity of the short-axis diameter ≥ 1 cm was even worse, only 19%, although the specificity was 97%. Takahashi and colleagues reported that the high proportion of adenocarcinoma cases in their study may have explained the low sensitivity of the conventional size criterion because lymph node metastasis of adenocarcinoma tends to arise even in small lymph nodes [6]. The proportion of adenocarcinoma cases in the metastatic group in this study was high, 78%, which was higher than those of previous reports (25–71%) [13–16]. This high proportion of adenocarcinoma cases could be one of the reasons for the low sensitivity of the conventional short-axis diameter evaluation. The increasing proportion of adenocarcinomas has been noted not only in research but also in reality in many countries [17, 18]. In addition, with innovations in imaging systems and prevalence of imaging screening tests, the proportion of early-stage lung cancer compared with those of other stages has gradually increased, which could cause microscopic metastasis rather than bulky metastasis. For these reasons, indices other than the short-axis diameter are needed more than before, and the volume, the short-axis/long-axis ratio, and the new index of sphericity were measured and analyzed in this study.

In this study, we introduced a new morphologic index, sphericity with 3D-CT, to evaluate lymph nodes sterically. Sphericity was previously applied to evaluate the changing shape of pebbles flowing down a river [11] and represents steric roundness. Sphericity of lymph nodes is calculated by dividing the surface area of the node by the surface area for a virtual sphere whose diameter is the largest diameter of the lymph node. Sphericity basically varies from 0 to 1, and the larger the sphericity, the greater its closeness to a sphere, with sphericity indicating 1 as the perfect sphere. In this study, the sphericity of metastatic lymph nodes was higher than that of nonmetastatic lymph nodes, which showed that metastatic lymph nodes became sterically round in an objective manner. Previous reports have described the round shape of metastatic lymph nodes. Fujiwara and colleagues reported subjective roundness using endobronchial ultrasound sonography in patients with lung cancer [19]. However, subjective assessment could be not only inaccurate, but also nonreproducible in terms of deciding the cutoff value. Bayanti and colleagues reported that the round shape index on horizontal CT showed metastatic lymph nodes in patients with lung cancer [20]. As their analysis was only of the axial CT section of the largest axial section of lymph nodes, they could have missed the characteristics of lymph node morphology in other directions. Because the sphericity index introduced in our study enables evaluation of all the aspects of a lymph node as a 3D object, the results may be considered to reflect the whole lymph node and to be more precise. Therefore, this is the first report objectively showing in 3D the spherical tendency of metastatic lymph nodes in lung cancer patients.

The AUC of 2D and 3D parameters were analyzed with the ROC curve. Although the short-axis diameter and the volume of lymph nodes are common in terms of evaluation of their size, the AUC of the former was higher than that of the latter. This was consistent with the findings of a previous report [6]. This might be a limitation of the resolution provided by 1-mm CT sections. Similar to size analysis, the sphericity and short-axis/long-axis ratio of lymph nodes are common in terms of the evaluation of their shape, and the AUC of the former was higher than that of the latter. Therefore, in this study, we combined the short-axis diameter and sphericity, which were superior indices of size and shape.

In the current study, when sphericity was applied to lymph nodes with short-axis diameters greater than or equal to 5 mm, the sensitivity of the sphericity for lymph node metastasis was higher than that of the conventional size criterion, i.e., short-axis diameter ≥ 1 cm, and had acceptable specificity. The scatterplot (Fig. 4) showed that most of the lymph nodes with short-axis diameters smaller than 5 mm were nonmetastatic regardless of the sphericity. However, among these small nodes, there were some nonmetastatic nodes with large sphericity. Several reasons are possible for the difficulty in applying sphericity to small-sized lymph nodes. Firstly, the resolution of 1-mm-section CT data might not have provided enough data for the sterical analysis of small-sized lymph nodes. Secondly, even small-sized lymph nodes without metastasis tend to be round, as small-sized lymph nodes may not be affected by compression from the surrounding organs. On the other hand, nonmetastatic lymph nodes of significant size tend to be flat-shaped, as their elasticity is high and they are easily compressed by the surrounding organs. However, once metastasis develops in the lymph node, its elasticity would be reduced, resulting in the spherical shape change. Although the sphericity could not be applied for small-sized lymph nodes, the incidence rate of lymph node metastasis to small-sized lymph nodes is low, as shown in Fig. 4. Therefore, sphericity could be a practical indicator for metastasis of lymph nodes of significant size.

Several limitations of this study should be acknowledged. First, this investigation was a retrospective one conducted at a single institute and with a relatively small number of patients. It is necessary to conduct a prospective multicentric study with PET CT and/or diffusion-weighted MRI [21] to improve the reliability of the sphericity in the diagnosis of lymph node metastasis, which is our next focus. Second, it is necessary to use 3D-CT software and half manual calculation to analyze sphericity, which could be minor obstacle in daily clinical practice. It is desirable that the function calculating the sphericity is equipped with the 3D-CT software working with a picture archiving and communication system (PACS). Finally, we did not include positron emission tomography (PET). In clinical practice, PET has been used in many institutions for lymph node evaluation. Although PET has been reported to have high sensitivity in predicting lymph node metastasis, its specificity is not satisfactory owing to the false-positives caused by inflammation [22]. Therefore, we focused on morphologic evaluation in this study, but we expect that integrating metabolic and morphologic evaluation will enable more accurate evaluation, which is a topic for future work.

## Conclusions

In conclusion, 3D-CT evaluation showed that metastatic lymph nodes became spherical. When sphericity was applied to lymph nodes of significant size, i.e., short-axis diameter ≥ 5 mm, it showed high sensitivity and specificity that could be applied in clinical practice.

## Data Availability

The data used and analyzed during the current study are available from the corresponding author on reasonable request.
